# The extended Infant Feeding, Activity and Nutrition Trial (InFANT Extend) Program: a cluster-randomized controlled trial of an early intervention to prevent childhood obesity

**DOI:** 10.1186/s12889-016-2836-0

**Published:** 2016-02-18

**Authors:** Karen J. Campbell, Kylie D. Hesketh, Sarah A. McNaughton, Kylie Ball, Zoë McCallum, John Lynch, David A. Crawford

**Affiliations:** Deakin University, Centre for Physical Activity and Nutrition Research, School of Exercise and Nutrition Sciences, Faculty of Health, Victoria, Australia; Department of Pediatrics, University of Melbourne, Victoria, Australia; School of Public Health, University of Adelaide, South Australia, Australia

**Keywords:** Randomized controlled trial, Infant, Early-childhood, Obesity, Prevention, Intervention, Scalability, Feeding, Physical-activity, Screen-time

## Abstract

**Background:**

Understanding how we can prevent childhood obesity in scalable and sustainable ways is imperative. Early RCT interventions focused on the first two years of life have shown promise however, differences in Body Mass Index between intervention and control groups diminish once the interventions cease. Innovative and cost-effective strategies seeking to continue to support parents to engender appropriate energy balance behaviours in young children need to be explored.

**Methods/Design:**

The Infant Feeding Activity and Nutrition Trial (InFANT) Extend Program builds on the early outcomes of the Melbourne InFANT Program. This cluster randomized controlled trial will test the efficacy of an extended (33 versus 15 month) and enhanced (use of web-based materials, and Facebook® engagement), version of the original Melbourne InFANT Program intervention in a new cohort. Outcomes at 36 months of age will be compared against the control group.

**Discussion:**

This trial will provide important information regarding capacity and opportunities to maximize early childhood intervention effectiveness over the first three years of life. This study continues to build the evidence base regarding the design of cost-effective, scalable interventions to promote protective energy balance behaviors in early childhood, and in turn, promote improved child weight and health across the life course.

**Trial registration:**

ACTRN12611000386932. Registered 13 April 2011.

## Background

Childhood overweight and obesity are prevalent across developed and developing nations [1, 2]. While prevalence rates may be slowing in some countries [3], the prevalence of overweight and obesity in children in lower socioeconomic environments appears to have increased [4]. Childhood overweight and obesity remain high priorities for public health and it is imperative that we address prevention comprehensively, including through the design of programs that can be scaled up [5]. Key to scalability are issues of intervention dose (timing, intensity and duration) and the capacity to utilize existing infrastructures [6]; both will determine future cost, and hence, sustainability. Determining the minimal dose of intervention needed for sustained change to children’s obesity risk behaviours, and in turn body weight, underpins the rationale for this extended version of an established intervention embedded within existing health services in Victoria, Australia.

Similar to other developed nations [1], in Australia ~25% of children aged 2–17 are overweight or obese [7]. The expression of adiposity begins in early life with nearly 23% of Australian 2–4 years affected [7]. Overweight and obesity are recognized to have negative consequences for children’s health and wellness during childhood and through to adult life [8, 9]. Further, the timing of weight gain is considered important. Evidence highlights that rapid weight gain across the first two years of life is strongly predictive of later adiposity in both childhood and adolescence [10]. It is important therefore that we acknowledge both the early expression of overweight and obesity and that the early years provide a vitally important opportunity for prevention.

Obesity-promoting lifestyle behaviours are established early. For example, Australian [11, 12] and international data [13–15] report that from an early age children are consuming diets high in energy-dense foods/drinks and low in fruits and vegetables. Our own data from the Melbourne Infant Feeding Activity and Nutrition Trial (InFANT) Program of children aged 9 and 18 months of age showed the consumption of energy-dense, nutrient-poor foods occurs as early as nine months of age with 12% of dietary energy provided by non-core foods [16]. There is also evidence of high levels of sedentary behaviour in early childhood. For example, a systematic review found that the average duration of television viewing for children under the age of two years reported by studies ranged between half an hour and more than five hours per day [17]. Data from the Melbourne InFANT Program shows that television viewing increases across the early childhood period [18]. While little physical activity data exists for children under two years, our research shows that 19-month old children spend an average of 184 min in light-intensity physical activity and 47 min in moderate- to vigorous-intensity physical activity each day, with the remainder of their day spent sedentary [19].

The early establishment of obesity promoting behaviours is important both because they will determine weight gain trajectories but also because these behaviours are known to track. For example, there is evidence of tracking of children’s dietary [16, 20, 21] and physical-activity [22, 23] behaviours from childhood to adolescence and adulthood. Early childhood thus provides a unique and circumscribed opportunity in which we might reduce risk of lifetime adiposity; a time within which to seek to establish lifestyle behaviours that will promote health and minimize the risk of the development of obesity and associated co-morbidities throughout life.

Children’s eating, physical-activity and sedentary behaviours are learnt and sustained in the home and evidence from our own [24] and others’ studies suggest this environment may improve children’s weight and energy-related behaviours [25, 26]. Parents shape children’s emerging food and physical activity choices through a variety of means including: their knowledge regarding eating, physical activity and sedentary behaviours; parenting style and feeding style; modelling of eating/activity; the food/facilities made available and accessible; food portion sizes and the use of food as rewards [27].

Several existing randomized controlled trials (RCTs) report that parent-focused interventions from birth hold promise for early childhood obesity prevention, with trials reporting modest effects on early childhood weight [26], diet [24–26] and sedentary behaviours [24]. In addition, results from a prospective meta-analyses incorporating these RCTs (Early Prevention of Obesity in CHildhood (EPOCH) Trial *n* = 2196) provides further support for this focus on interventions in early life [28]. In that analysis, compared to controls, intervention children at age 18 to 24 months had a significantly reduced zBMI, were breastfed for longer, spent less time viewing television, and were significantly less likely to be exposed to a range of obesity-promoting feeding behaviours, specifically controlling feeding style, use of food as a reward, and pressure to eat [29].

However, while these pooled data showed important effects on zBMI, three of the four constituent studies have recently reported that there were no differences in zBMI between intervention and control groups at age five [30–32]. The prospective meta-analyses will be undertaken to confirm this loss of intervention effect over time when the last of the four trials [33] collects their 5-year old post-intervention data (2016). This potential failure to maintain intervention effect is perhaps not surprising given the complexity and the dynamic nature of the targeted energy-related behaviours across early childhood. The substantial developmental change occurring in the early years frequently heralds a particularly challenging period for parents. For example, parents report increased rejections of food and proposed limits to screen time [34] as their child moves through the toddler years. It is likely that parents will require ongoing support to develop strategies that can address the evolving challenges they face.

One of these RCTs, the Melbourne Infant Feeding Activity and Nutrition Trial (InFANT) Program (herein referred to as the Melbourne InFANT Program), informed the protocol for the current study, known as the InFANT Extend Program. The methodology employed for the Melbourne InFANT Program have been previously published [35]. In brief, the Melbourne InFANT Program was a cluster-randomized controlled trial of a community-based, early obesity prevention program, designed to be integrated into existing service delivery systems. The intervention comprised 6 × 2 h sessions delivered quarterly to first-time parents from when infants were approximately three months of age to approximately 18 months of age. Sessions were delivered within existing first-time parent groups, established by community Maternal and Child Health nurses (MCHn) as part of the free universal health care system in Melbourne, Australia. These groups had, in previous years, continued without nurse facilitation for approximately 18-months [36]. The Melbourne InFANT Program commenced where the MCHn involvement with the groups ceased, and parents took over their own management of the groups. The Program sessions utilized anticipatory guidance, providing information and developing skills (in anticipation of their relevance), regarding what and how to feed, active play opportunities, alternatives to screen time and restraint, and parent modelling of healthy eating, physical activity and reduced sedentary behaviours. The group format promoted discussion of strategies, successes and overcoming barriers to key messages. The control group received usual care as well as quarterly newsletters (six in total) on general child health topics not related to obesity-promoting behaviours.

Strengths of the Melbourne InFANT Program included: comprehensive assessment of targeted behaviours using gold standard methods (objective assessment of body mass index (BMI), physical activity and sedentary time, and three non-sequential 24-h dietary recalls); use of existing social groups to potentially facilitate, support and increase intervention dose by non-facilitated contacts between sessions; scalability - as the program was developed to be both low dose and community-based, allowing feasible transfer into existing public health infrastructures; high recruitment and retention rates and incorporation of an economic evaluation [24]. The Melbourne InFANT Program has been adapted for use within eight local government areas in Victoria, Australia and translation of this program from RCT to community use is currently being evaluated.

### The InFANT Extend program

The current study builds on the early outcomes of the Melbourne InFANT Program [24, 35]. This cluster randomized controlled trial will test the efficacy of an extended (33 versus 15 month) and enhanced (use of web-based materials, and Facebook® engagement), version of the original InFANT intervention in a new cohort. Outcomes at 36 months of age will be compared against the control group.

#### Primary outcomes

In comparison to the control group children, the intervention group children at 36 months of age will exhibit lower body weight and reduced waist circumference.

#### Secondary outcomes

In comparison to the control group infants, the intervention group infants at 18 and 36 months of age will:

- Consume more serves of fruits and vegetables, and fewer serves of sugar-sweetened beverages and energy-dense snack foods;
- Spend more time being physically active and less time in sedentary behaviours, specifically television viewing.
- Exhibit improved energy-related lifestyle patterns (combining measures of diet, physical activity and screen time)

In comparison to the control group parents, the intervention group parents (when child is 18 and 36 months of age) will demonstrate:

- Greater knowledge regarding infant eating, physical activity and sedentary behaviours and more positive attitudes /beliefs regarding their capacity to influence these behaviours;
- Greater adoption of desired feeding strategies, including parental modelling of healthy eating, the division of responsibility in feeding, and increased availability of promoted (targeted) foods in the home;
- Greater adoption of strategies, including modelling, for increasing opportunities for physical activity and reducing opportunities for sedentary behaviours.

## Design and Methods

### Overall study design

This study will enable assessment of the effectiveness of a 33-month parent-focused child obesity-prevention intervention (compared with a no-intervention control).

Ethical approval was granted by Deakin (EC-175-2007 (Part 2- 2007–175) and the Department of Education and Early Childhood Development (Victoria, Australia) (2011_001000). This trial is registered with the Australian New Zealand Clinical Trials Registry (ANZCTR 12611000386932).

### Study participants and recruitment

The recruitment process for the InFANT Extend Program will largely replicate that used in the original Melbourne InFANT Program [24, 35]. An important exception relates to the local government area (LGA) recruitment strategy. To seek to address the over representation of university educated women in the Melbourne InFANT Program [24] seven relatively disadvantaged Victorian LGAs will be purposively recruited. These LGAs will selected by the group level variable *Index for Socio Economic Disadvantage* (IRSD) [37] such that all will be in the lowest tertile of disadvantage (i.e. most disadvantaged). It is important to note that there will be distinct areas of greater and lesser socioeconomic advantage within each LGA and these are indicated by the IRSD of postcode regions within each LGA. For practical reasons, each LGA will sit within a 75km radius of the research center (Geelong, Victoria, Australia).

Eighty percent of eligible first-time parents’ groups (rounded to next even number to ensure equal within LGA allocation to control and intervention groups) within each of these LGAs will be randomly selected and approached by research staff for recruitment during one of the standard nurse-facilitated group sessions. Individual parents will be eligible to participate if they give informed written consent, are first-time parents and are literate in English. Infants with chronic health problems likely to influence height, weight, levels of physical activity or eating habits will be excluded from analyses but will be permitted to participate in the program.

Parent groups will be eligible if eight or more parents choose to enroll in the study. To facilitate inclusion of participants experiencing disadvantage, groups commencing in MCH centres considered relatively socioeconomically disadvantaged (as determined by the post code of the region within each LGA), will be eligible if six or more parents enroll. When first-time parents’ groups decline to participate, the next randomly selected group within the LGA will be approached. Non-consenting parents within participating groups will be permitted to attend the intervention sessions, but will not be required to provide data or be contacted by the research team in any other way.

Randomization of first-time parents’ groups (clusters) to intervention or control will occur after recruitment to avoid selection bias [38]. Randomization (stratified by LGA) will be conducted by an independent statistician. While parents will not be blinded to allocation, they will not be informed of the study aims or hypotheses and the recruiting emphasis will focus on promoting healthy eating and active play from the start of life. Staff measuring height and weight will not be blinded to intervention status as they will deliver the intervention, however, data entry and analyses will be conducted with staff blinded to participant’s group allocation.

### Sample size

#### Power and sample size

Child weight and waist circumference are the primary outcome measures for this study and considered the most difficult outcomes to change. Secondary aims of this intervention are to increase children’s fruit and vegetable consumption and time spent physically active, and to decrease consumption of sweetened drinks and time spent sedentary.

Australian national data [39] report an average weight and waist circumference in 3-year old children of 16.4kg (SD = 2.2kg), and 51.1cm (SD = 3.8cm) respectively [39]. Further, these data report that at this age Australian children consume an average of 85g (SD = 82g) of vegetables (not including potato), 202g (SD = 129g) of fruit and 78mL (SD = 121mL) of sweetened drinks daily. Additionally this group is reported to spend an average of 117min (SD = 30min) being active and 662min (SD = 71min) being sedentary daily [40].

To detect a 5% difference in weight between groups at age three (reducing average weight in the intervention group by approximately 1kg), with Type I and Type II errors of 5% and 20% respectively, the number of subjects required is given by:

$$ \mathrm{N}\ \left(\mathrm{number}\ \mathrm{of}\ \mathrm{subjects}\right) = 32/\mathrm{E}\mathrm{S}\ {\left(\mathrm{Effect}\ \mathrm{S}\mathrm{ize}\right)}^2 $$

$$ \mathrm{E}\mathrm{S} = \left(\mathrm{mean}\ \mathrm{x}\ \%\ \mathrm{meaningful}\ \mathrm{difference}\right)/\mathrm{standard}\ \mathrm{deviation} $$

$$ \mathrm{So},\ \mathrm{N} = 32/0.382 = 223 $$

This sample size will also allow us to detect differences of: 4% in BMI (i.e. 0.66kg/m^2^), 3% in waist circumference (i.e. 1.5cm), 36% in vegetable consumption (i.e. 31g, equivalent to approximately ½ a serve/day); 24% in fruit consumption (i.e. 49g, equivalent to approximately 1/3 of a serve/day); 60% in sweetened drink consumption (i.e. 47mL/day); 10% in active time (i.e. 12min/day) and 4% in sedentary time (i.e. 26min/day).

As this study will randomize by first-time parents groups, we need to take account of within-group clustering and increase our sample size according to the design effect/inflation factor (DEFF). The design effect is given by: DEFF = 1 + [(n-1) x ICC], where n is the number of people in each cluster and the ICC is the intra-class coefficient. Based on our previous experience working with first-time parents groups, we estimate that each cluster contains an average of nine mother-infant pairs, and the ICC is estimated to be 0.1 (based on data from the Melbourne InFANT Program), thus the design effect is 1.8. We will also adjust our sample size to account for estimated attrition over the three years of the study (<25%).

Therefore, our final sample size is: (223 x 1.8) / 0.75 = 535. To achieve an equal number of groups in each arm of the trial (mean number of participants in groups = 9), we will aim to recruit 540 participants from a total of 60 first-time parents’ groups (30 groups and 270 participants in each arm of the trial).

**Fig. 1:**
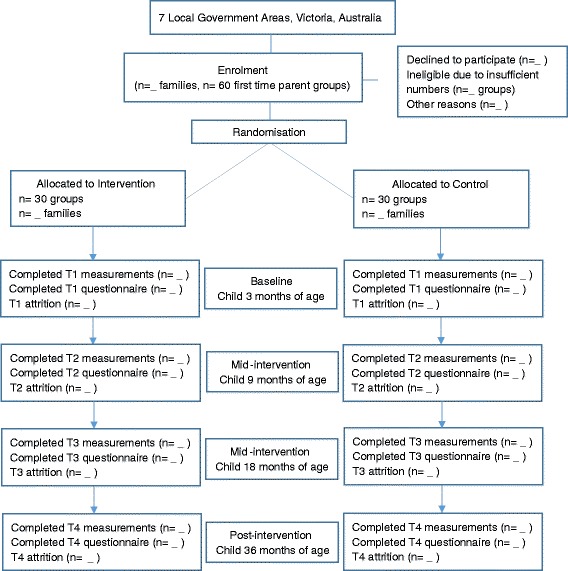
CONSORT flow diagram

The CONSORT flow diagram is outlined in Fig. 1.

### Intervention group

The intervention arm will receive the previously trialed Melbourne InFANT Program delivered in first-time parent groups until child is aged 18-months. The intervention content has been reported elsewhere [24, 35] and is outlined in the background in this paper. In the current InFANT Extend Program, the Melbourne InFANT Program content will be largely replicated however some enhancement, informed by both quantitative [24] and qualitative [41] outcomes will be incorporated (e.g. parent's desire for more practical strategies for preparing food and playing with children). To reflect the transition in technology preference of parents over the time since the Melbourne InFANT Program was trialed (2008–2010) we plan to alter some aspects of program delivery. While the original Program content was delivered in groups with the aid of a DVD (which parents took home), in the present study DVDs will no longer be used and all content will be made available online via dedicated webpages. The use of webpages provides opportunities previously not afforded, to monitor website use and thus inform at a group level, the dose of intervention received.

In addition, during the first 18 months, the original Melbourne InFANT Program will be extended by the addition of first-time parent group Facebook® (Menlo Park, CA, USA) Pages. On-line engagement will be restricted to the individual first-time parent group, and will be mediated by the group facilitator, a nutrition expert, for up to one hour per week. This use of social media is anticipated to promote parent engagement between their regular social meetings, and to provide opportunities to: share their child feeding and activity related outcomes (knowledge, questions, successes, challenges); enable the group facilitator to provide support; and to reiterate program messages.

As in the original Melbourne InFANT Program, parents will be mailed one newsletter each quarter. These newsletter were well received in the original program and provided an important opportunity, given the infrequency of intervention sessions (three monthly), to engage mothers and to reinforce key program messages. This aspect of the program will be modified and extended in the InFANT Extend Program and will form the key focus of the extended intervention. Group delivery beyond 18 months is not feasible given we know that first time parent groups – the site of delivery - are often not sustained after this time [36]. Furthermore, our previous experience highlighted that while attendance to 18 months was relatively high, attendance figures began to decline at around 12 months of age. Therefore, in the current study, the Melbourne InFANT Program content will be reiterated and extended through delivery of intervention messages via six emailed newsletters (3 monthly from child age 18 months to 3 years). Newsletters will contain web links directing participants to specifically designed content within the Melbourne InFANT Program website (discussed below). This content will seek to reinforce and build upon skills and knowledge developed in the group-delivered intervention. It will also introduce new knowledge and skills known to be of relevance to parents in the promotion of healthy eating and physical activity behaviors across the often behaviorally challenging toddler years. In addition, participants will be reminded of newly developed toddler focused content on the Program website through monthly emails and Facebook® posts. The focus of each intervention session (3–18 months) and each newsletter provided in the extension (21–36 months) is outlined in Table 1.

**Table 1:** Intervention time-frame and focus for the InFANT Extend Program

Infant age	Emerging behaviours	Anticipatory guidance intervention focus (group, newsletters, Facebook® to 18 months; newsletters and Facebook® 18–36 months)
3mo	Early weaning and introduction of solids. Introduction of nutrient poor foods. Parents development of a feeding style	To support parents to delay weaning/introduction of solids to around 6 months To provide basic principles related to best practice in early feeding To introduce basic concepts regarding parental feeding styles and how these might relate to beliefs about parenting.
6mo	Adoption by parents of a feeding style and TV viewing habits Food rejection by infants	To develop parents’ understanding regarding: *feeding styles and impact on children’s eating *basic nutrition principals * sedentary behaviours in families To introduce national recommendations for no screen exposure (television viewing) until 2 years of age and reasons for this To develop parents’ understanding of ‘normal’ food rejection and how to interpret and manage
9mo	Increasing use of TV Parents’ increased awareness of child mobility. Infant crawls and pulls self upright and walks with handhold	To develop understanding regarding: * parental modeling of eating, sedentary and physical activity behaviours * impact of eating, activity and sedentary behaviours on health of children and adults and the provision of opportunities to promote healthy eating and engagement in play
12 mo & 15 mo & 18 mo	Increasing autonomy of child in eating and activity Infant stands without support and beginning to walk	Continued development of themes/skills regarding: * eating and moving for health – parents and children * how to feed/how to manage food rejection and demands * providing fail-safe food and activity environments
21,24,27,30,33,36 mo	Child independence in activity and feeding; desire to be in control and to choose	Continued development of themes/skills regarding: * eating and moving for health – parents and children * how to feed/how to manage food rejection and demands Practical strategies for incorporating more active play into family routines Development of fundamental movement skills through everyday play * providing supportive food and activity environments

Key: mo - months

### Control group

The control group families will receive usual care from their MCHn. In addition, these families will be sent general health newsletters (e.g. dental health, sun protective behaviours, general safety) every three months across the child’s first three years (11 newsletters in all). Consistent with the intervention group, control group participants will receive birthday and Christmas cards. These families’ participation will be acknowledged with gifts (to a maximum value of $15.00) on receipt of completed questionnaires.

### Data collection

#### Measures

As outlined in Table 2, parent and infant data will be collected at 3, 18 and 36 months. Standard demographic and socio-economic information will be collected by parental report at baseline (3 months). Additional measures to be collected are detailed below.

**Table 2:** Measures and time-frame for the study

Intervention timeline - year		1	2				3	4
Measures		Infant age						
		3 months	6 months	9 months	12 months	15 months	18 months	36 months
Dietary intake	Parent Child	✓ ✓		✓*			✓ ✓	✓ ✓
Sedentary behaviour	Parent Child	✓		✓ ✓			✓ ✓	✓ ✓
Physical activity	Parent Child	✓		✓ ✓			✓ ✓	✓ ✓
Family food environment	Parent	✓		✓			✓	✓
Family physical activity & sedentary environment	Parent	✓		✓			✓	✓
Demographic data	Parent	✓						
Anthropometric data	Parent Child	✓ ✓		✓ ✓			✓ ✓	✓ ✓

*Modified FFQ

#### Primary measures

##### Child’s anthropometry

Height, weight and waist circumference will be measured by study staff who will undergo training with a paediatrician specialized in clinical nutrition. Recumbent length (in infants) will be assessed using a calibrated length mat/height and from standing age will be assessed using a calibrated stadiometer. Waist circumference (minimum circumference between the rib cage and iliac crest) will be measured using a non-stretchable tape measure at 18 and 36 months of age. BMI z score will be calculated using WHO growth standards [42].

#### Secondary measures

##### Child’s dietary intake

Children’s dietary intake will be assessed at 18 months and 3 years with a parent completed food frequency questionnaire. This 79 item FFQ has been used within the original InFANT Program and is currently being validated against the three days of 24 h recall also collected in that study when children were 18 and 36 months of age. Data will be analyzed using an in-house, specially designed database using the 2007 Australian Food and Nutrient Database (AUSNUT) Database [43]

### Measurement of physical activity and sedentary behaviours

Seven days of objectively assessed physical activity data will be collected using accelerometers at 18 and 36 months. At this time children will be fitted with an ActiGraph accelerometer which they will wear for eight consecutive days (which will capture weekday and weekend day activity and sedentary patterns) [44]. ActiGraph monitors are small, light and unobtrusive and are worn on a belt around the waist. This methodology was successfully employed during the original InFANT Program intervention when children were aged approximately 19 months. ActiGraph counts correlate (up to r = 0.70) with energy expenditure estimated by direct observation and doubly-labelled water respectively, among 3–5 year old children [45, 46] and correlate highly (r = 0.87) with energy expenditure estimated by indirect calorimetry among children [47]. Counts will be recorded at 15-s epochs to accurately capture the sporadic and intermittent activity patterns of young children. Data will be downloaded and then reduced to total counts/day, minutes/day and percentage of time spent sedentary, and in light-, moderate- and vigorous-intensity physical activity using age-appropriate cut points [48]. In addition, indirect measures of children’s physical activity will be assessed by parental report including: parental engagement in physically active play and the number of hours the child typically spends playing outdoors on weekdays and weekend days.

In addition, parents will be asked to indicate how much time (hours/min) their child usually spends watching television/DVD and playing electronic games on a typical weekday (Monday-Friday) and on a typical weekend day (Saturday and Sunday) and to estimate the amount of time spent in situations that restrict movement (e.g. stroller, playpen) at 3, 9, 18 and 36 months. Test-retest reliability of these items in a previous study ranged from ICC = 0.5–0.9 [49]. Parental reports of their child’s “usual” TV viewing has been shown to correlate with both videotaped observations of the child’s TV viewing [50] and with parental diaries of viewing [51].

### Parent’s diet

Parents’ dietary intake will be assessed using a validated Food Frequency Questionnaire, *The Cancer Council’s Dietary Questionnaire for Epidemiological Studies (Version 3.1)* when child is 3, 18 and 36 months of age [52].

### Parent’s physical activity and television viewing

Parents will report their physical activity behaviours using the validated *Active Australia Survey* [53] at 3, 18, 36 months. Parents will also report the total time they spend watching television during their leisure-time in a typical week [54].

### Home food environment

A range of home food environment variables will be assessed at 3, 18 and 36 months. Aspects of *nutrition knowledge* focused around nutrition targets of the intervention will be assessed using modified subscales of the validated *Nutrition Knowledge Questionnaire* [55, 56], Parent Feeding Style will be assessed using a modified version of *the Comprehensive Feeding Practices Questionnaire* [57]. Covert restriction will be assessed using a validated subscale [58].

Opportunities for *modelling of healthy eating* (e.g. sharing family meals) and *home food availability* will be measured using previously established tools [59].

Parental confidence regarding promoting healthy eating (and reducing sedentary time/promoting physical activity) will be assessed using established tools [60].

### Home physical activity and sedentary environment

Parents will be asked general questions relating to their knowledge about physical activity in early childhood, their interactions with their child around physical activity and an audit checklist on the physical activity and sedentary home environment at 3, 18, 36 months.

### Economic evaluation

#### Health service use

Exposure to the InFANT Extend Program may have implications for families’ use of the broader health system if the information provided through the program reduces parent’s help-seeking behaviour elsewhere. It is therefore important to monitor use of relevant health services, especially MCHn visits as the primary health provider in this population. Parents will be asked to report the use of services related to their infant’s or their own weight, diet/eating behaviours or physical activity in order to capture any differential use of health and other services associated with the intervention. Parents will be asked to report specifically on their use of MCHns, and more generally on a broad range of services. In each case, parents will be asked to report the number of occasions of service use and, where applicable, any financial cost. The investment of resources involved in reported use of health services will be costed using established unit costs for wages, services and material costs in Australian dollars.

### Statistical analyses

Intervention effects will be assessed based on intention to treat principles and taking into account the cluster-based sampling design. Generalized Estimating Equations [61] using the xtgee function in Stata, will be used to fit longitudinal regression models enabling comparison of primary outcome variables between intervention and control groups, adjusted for baseline values where appropriate (infants were not consuming foods nor mobile at baseline, i.e. 3 months of age, hence adjustment for diet or physical activity variables is not possible).

## Discussion

The prevalence of overweight and obesity in early childhood remains high and is determined in part, by eating, physical activity and sedentary behaviours. These behaviours are predominantly learnt and supported in the home during the first few years of life, and are likely to influence health throughout life. Given this, the early years hold promise as a time when obesity prevention may be most effective.

While there is a growing body of evidence to support the proposition that family focused interventions can improve children’s energy-related behaviours and weight, there remain many unanswered questions regarding the dose (timing, intensity and duration) of intervention delivery, key issues for translation of interventions into real world settings. The issue of scalability is of fundamental importance. The current study builds upon the Melbourne InFANT Program which is currently being trialed in community settings across Victoria Australia. This opportunity for translation speaks to the program’s potential scalability. The translation (uptake, modification, facilitator and end user satisfaction) of the Melbourne InFANT Program is currently being evaluated. The InFANT Extend Program’s focus on the toddler years (18 months to three years) explores the opportunity to build on the Melbourne InFANT Program’s early support regarding energy balance behaviours (3–18 months of age) through the reiteration of key messages and the timely extension of this support through the introduction of new knowledge, ideas and skills across the toddler years.

In summary, this cluster-randomized controlled trial assessing the efficacy of a low dose, scalable, web-based addition to the existing Melbourne InFANT Program will provide important information regarding capacity and opportunities to maximize early childhood intervention effectiveness over the first three years of life. This study continues to build the evidence base regarding the design of cost-effective, scalable interventions to promote protective energy balance behaviors in early childhood, and in turn, promote improved child weight and health across the life course.

## Abbreviations

BMI: Body mass index
EPOCH: Early Prevention of Obesity in CHildren
FFQ: Food frequency questionnaire
InFANT: Infant Feeding Activity and Nutrition Trial
MCH: Maternal and Child Health
MCHn: Maternal and Child Health nurse
RCT: Randomized Controlled Trial
WHO: World Health Organisation
